# Ethnicity does not matter: Comparable reversed congruency effects for gaze stimuli from same- and other-ethnicity faces

**DOI:** 10.1007/s00426-025-02200-6

**Published:** 2025-10-29

**Authors:** Kenta Ishikawa, Mario Dalmaso, Yoshihiko Tanaka, Takato Oyama, Matia Okubo

**Affiliations:** 1 Department of Psychology, Faculty of Liberal Arts, Teikyo University, 359 Otsuka, Hachioji-shi, Tokyo 192-0395 Japan; 2 Department of Developmental and Social Psychology, University of Padova, Padova, Veneto Italy; 3 Department of Psychology, Senshu University, Kawasaki, Kanagawa Japan; 4 Faculty of Human Informatics, Aichi Shukutoku University, Nagakute, Aichi Japan

**Keywords:** Gaze, Social versus non-social, Spatial Stroop task, Reversed congruency effect, Ethnicity

## Abstract

In spatial Stroop tasks with gaze stimuli, the reversed congruency effect (RCE) refers to slower responses in congruent (i.e., a gaze pointing right presented on the right side of the screen) than incongruent (i.e., a gaze pointing right presented on the left side of the screen) trials. The nature of the RCE may stem from social attention mechanisms. In line with this social account, an increasing number of studies have shown that the RCE is modulated by characteristics of facial stimuli, such as social relevance or emotions. The present study investigated, through two cross-cultural online experiments, whether the RCE can be shaped by ethnicity. A total of 163 East Asian (Japanese) and European (Italian) participants completed a spatial Stroop task featuring East Asian and European faces. The two types of faces were presented either intermixed within the same block (Experiment 1) or in separate blocks (Experiment 2). The results revealed, in both experiments, a robust RCE, irrespective of participant or facial ethnicity. Overall, these findings offer new insights into the RCE and the boundary conditions for its potential modulation by social factors.

**Supplementary Information:**

The online version contains supplementary material available at 10.1007/s00426-025-02200-6.

## Introduction

In our daily lives, various directional cues guide our attention and actions. For example, the gaze of others reveals the direction of their interests or concerns during social interactions (Emery, 2000; Frischen et al., 2007), whereas road signs indicate the paths that we should follow while driving. These directional cues are broadly classified as social or non-social cues based on their characteristics and contexts. By definition, social stimuli (such as eye gaze) elicit social attention, which reflects the interests and intentions of others (Friesen & Kingstone, 1998; Ristic & Kingstone, 2005), whereas non-social stimuli (such as an arrow) do not (Friesen et al., 2004). An enormous number of studies have been conducted to clarify the attentional characteristics of social and non-social stimuli, with ongoing debates about their similarities and differences (see a review by Chacón-Candia et al., 2022).

Recent studies have demonstrated a unique attentional phenomenon for eye-gaze stimuli, known as the reversed congruency effect (RCE) elicited by a spatial Stroop task (e.g., Cañadas & Lupiáñez, 2012; Marotta et al., 2018). The classic version of the spatial Stroop task used in this context typically involves identifying the direction of an arrow (e.g., left or right) while ignoring its spatial location (e.g., left or right). Normally, reaction times (RTs) are slower, and accuracy decreases, in incongruent trials (e.g., a left-pointing arrow appearing on the right side of the screen) compared to congruent trials (e.g., a left-pointing arrow appearing on the left side of the screen). However, when eye-gaze stimuli are used rather than arrows, this pattern is reversed: RTs are slower, and accuracy decreases, for congruent trials than for incongruent trials (i.e., the RCE; Cañadas & Lupiáñez, 2012; Marotta et al., 2018).

Three main social accounts have been proposed to explain RCE, namely the eye-contact hypothesis (Cañadas & Lupiáñez, 2012; Marotta et al., 2018), the joint attention hypothesis (Edwards et al., 2020), and the joint distraction hypothesis (Hemmerich et al., 2022). The eye-contact hypothesis proposes that in incongruent trials, the gaze is directed toward the observer. This perception of direct eye contact is socially salient and facilitates responding, leading to faster RTs in incongruent than in congruent trials (Cañadas & Lupiáñez, 2012; Marotta et al., 2018). According to the joint attention hypothesis (Edwards et al., 2020), the RCE occurs because incongruent gaze trials create episodes of shared attention. Specifically, when a face is presented on either the left or right side and looks toward the fixation point, its gaze appears to align with the observer’s focus of attention at the centre of the screen. As a result of the facilitation effect of joint attention, RTs in incongruent trials become shorter, leading to the presence of the RCE. Finally, the joint distraction hypothesis proposes that in congruent trials, participants’ attention is drawn outside the task-relevant area of the screen (Hemmerich et al., 2022). As a result, it takes additional time to shift attention back to the gaze stimulus, which increases RTs in congruent trials relative to incongruent ones, leading to the presence of the RCE. Considered together, these three accounts highlight different ways in which social factors may contribute to the emergence of the RCE. The social account for RCE is also supported by increasing evidence showing that various social factors—such as facial expressions, socio-cognitive development, and perceived intentionality—can influence the strength of the RCE (Aranda-Martín et al., 2022; Ishikawa et al., 2021, 2025; Jones, 2015; Marotta et al., 2022; Tanaka et al., 2023). In this regard, Jones (2015) showed that the RCE for gaze stimuli was enhanced for emotional expressions such as anger and happiness compared to neutral faces, whereas Ishikawa et al. (2025) demonstrated that it was elicited by human but not by robot faces, likely reflecting differences in perceived social intentionality.

However, several other studies suggest that social accounts alone may not be sufficient to fully capture the nature of the RCE, as this effect has been shown to be modulated even by non-social factors, such as the perceptual complexity of the stimulus (Román-Caballero et al., 2021a, b; Tanaka et al., 2024). For example, Román-Caballero et al. (2021a) reported that the RCE occurred even for non-social stimuli like arrows when the perceptual complexity of their background was increased. Moreover, Tanaka et al. (2024) showed that tongue stimuli, which can be categorised as non-social direction stimuli, produce the RCE. They argued that perceptually complex stimuli, which hinder the rapid identification of stimulus direction, delay early perceptual processing, such as target-background segregation. This delay alters the temporal dynamics of stimulus-driven response activation and later-developing response inhibition, ultimately producing the RCE. Consistent with this argument, no RCE was observed when using cartoon eyes, which were assumed to facilitate the extraction of gaze direction from the background (Chen et al., 2022). Overall, these findings enrich the interpretation of the RCE by suggesting that it may arise from an interplay of both social and perceptual mechanisms.

### Testing the influence of ethnicity on the RCE

As outlined above, two primary accounts—the social and the non-social—have been proposed to explain the RCE (Aranda-Martín et al., 2022; Dalmaso et al., 2023; Edwards et al., 2020; Hemmerich et al., 2022; Ishikawa et al., 2021, 2025; Marotta et al., 2018, 2019; Román-Caballero et al., 2021a, b; Tanaka et al., 2024, 2025). To further explore the potential social underpinnings of the RCE, the present study used a spatial Stroop task involving faces and participants of two different ethnicities: East Asian and European.

Ethnicity is a fundamental social dimension, deeply involved in various mechanisms underlying face processing. For instance, it is well established that individuals experience greater difficulty in perceiving facial characteristics (e.g., facial expressions, age, gender, and mental state estimation) in individuals of different ethnic groups compared to those of their own (Adams et al., 2010; Elfenbein & Ambady, 2002; Elfenbein et al., 2007). Moreover, various effects of ethnicity on gaze perception have also been documented, including gaze-mediated orienting of attention (Dalmaso et al., 2015; Zhang et al., 2021, 2023), sensitivity to gaze direction (Collova et al., 2017; Uono & Hietanen, 2015), and selective attention to the eye region (Kawakami et al., 2014). In this regard, Pavan et al. (2011) observed a reduced gaze-mediated orienting of attention when European participants were presented with eye-gaze stimuli from African than European faces (for additional results, see also Dalmaso et al., 2015; Ward et al., 2025; Weisbuch et al., 2017). More important for the present study, Collova et al. (2017) conducted a gaze direction discrimination task using East Asian and European faces. Their findings indicated that sensitivity to gaze direction was higher for faces of the same ethnicity than for those of a different ethnicity, regardless of the participant’s ethnicity. Similar results were also reported by Uono and Hietanen (2015) who found that European participants were more accurate in discriminating gaze direction in same-ethnicity faces compared to East Asian faces. Interestingly, East Asian participants did not show this advantage, possibly reflecting cultural norms that discourage direct eye contact. These findings indicate that attentional processing of gaze can be prioritised when interacting with members of one’s own ethnic group, at least under certain circumstances. Accordingly, mechanisms such as joint attention and joint distraction (Cañadas & Lupiáñez, 2012; Edwards et al., 2020; Hemmerich et al., 2022; Marotta et al., 2018) may be less likely to be engaged for other-ethnicity faces, which could lead to a reduction or even absence of the RCE in cross-ethnic contexts. In contrast, the absence of modulation by ethnic membership may indicate that this variable does not substantially contribute to shaping the RCE, at least not beyond the influence already exerted by other factors (e.g., perceptual). To systematically examine these possibilities, we conducted two experiments using a spatial Stroop task in which we manipulated the ethnicity of target faces (East Asian and European) and included both East Asian (Japanese) and European (Italian) participants, thereby adopting a cross-cultural perspective. This methodological framework provided a comprehensive opportunity to investigate the potential social dimension of the RCE from a novel perspective, offering deeper insights into the interplay between ethnicity and eye-gaze processing. Arrow stimuli were also included as non-social control stimuli, for those we expected to observe a standard congruency effect (e.g., Cañadas & Lupiáñez, 2012; Marotta et al., 2018).

## Experiment 1

### Method

#### Participants

We recruited 82 participants, 40 of whom were East Asian (Japanese; 5 men, 34 women, and one nonbinary; *M*_*age*_ = 20.28 years, *SD* = 2.21) and 42 European (Italians; 19 men, 22 women, and one nonbinary; *M*_age_ = 26.21 years, *SD* = 2.83). East Asian participants were recruited in university classrooms for course credit, whereas European participants were recruited via the Prolific crowdsourcing platform and received €3 for their participation. We obtained informed consent from all participants.

To estimate the necessary sample size, we conducted a priori power analysis. We used G*Power 3.1 (Faul et al., 2007), assuming a significance level of α = 0.05 and an effect size of *d* = 0.5, as referenced in Jones (2015). The analysis indicated that a minimum of 34 participants per group would be required to achieve 80% power to detect effects. We therefore aimed to recruit approximately 40 participants for each ethnic group (East Asian and European). The study received approval from the University Human Research Ethics Committee (approval number 23-S001-1) and the Ethics Committee for Psychological Research (approval number: 4654). All experiments were conducted according to the principles outlined in the Declaration of Helsinki.

#### Material and stimuli

We used East Asian and European faces, as well as two black arrows, as target stimuli. The faces were obtained from the MR2 Database (Strohminger et al., 2016) and comprised two male and two female models (gazing leftwards and rightwards) for each ethnicity, for a total of 8 facial stimuli. The two arrows (both pointing leftwards and rightwards) were created to occupy about the same spatial region of eye-gaze stimuli. The experiment was developed in jsPsych 6.3.1 (de Leeuw, 2015).

#### Procedure

**Fig. 1:**
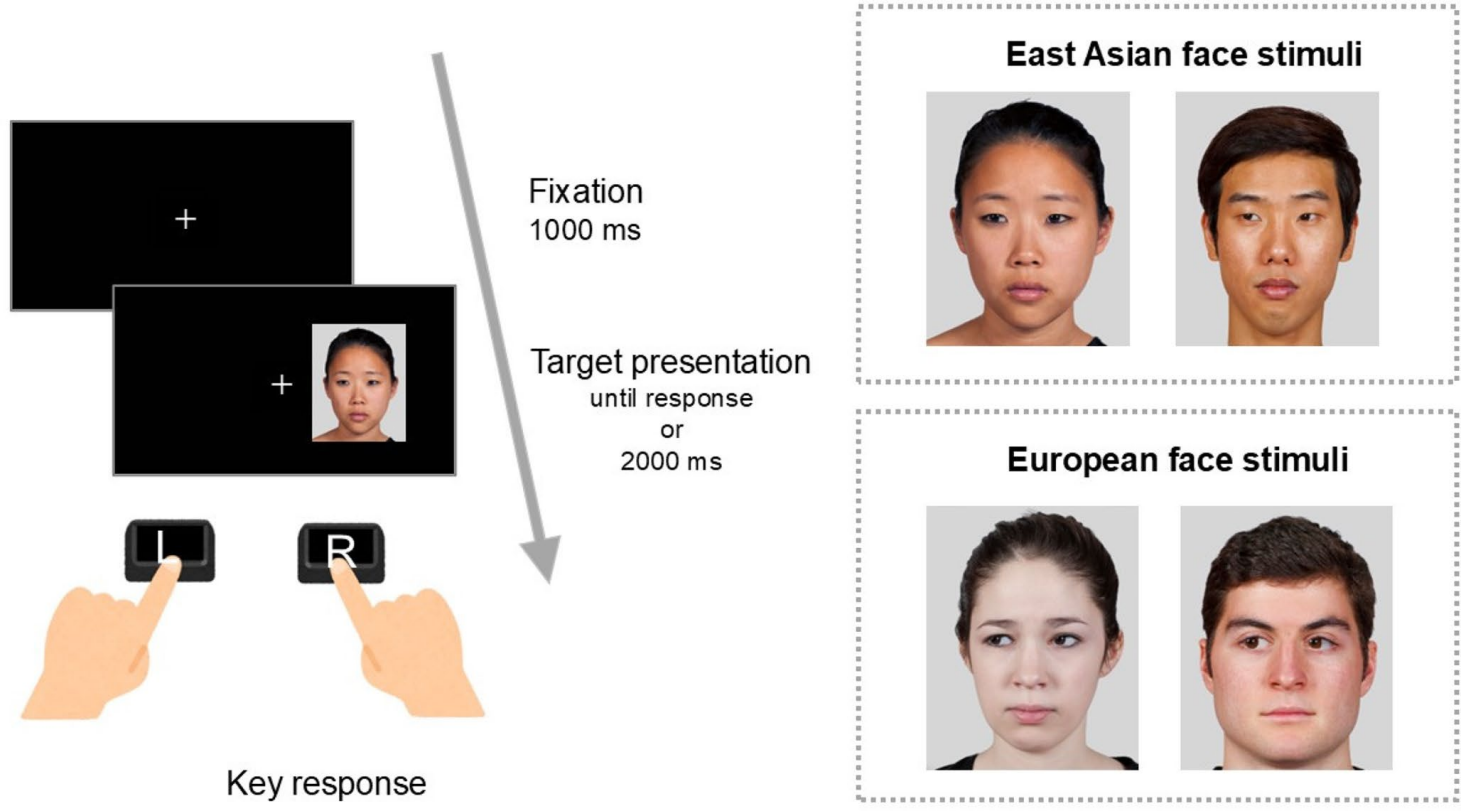
Examples of a trial sequence (left panel) and facial stimuli in the spatial Stroop task (right panel). The trial sequence moves from the top to the bottom. The example illustrates an incongruent trial with an East Asian face. In Experiment 1, faces were presented within the same block, whereas in Experiment 2, they were presented in separate blocks

The experiment was conducted online using the Pavlovia platform (Bridges et al., 2020). Participants were asked to conduct the experiment in a quiet room and to sit approximately 40 cm from the computer screen. During the task, they indicated the direction in which the target pointed (left or right) as quickly and accurately as possible while ignoring its location (left or right) on the screen. The task comprised 192 trials, with target types varying between arrows (64 trials), East Asian faces (64 trials), and European faces (64 trials), as well as congruency (congruent and incongruent). These trials were evenly divided into 96 congruent and 96 incongruent trials. Participants proceeded to the experimental trials after completing 12 practice trials (see Fig. 1).

At the beginning of each trial, a fixation cross was presented for 1000 ms at the centre of the screen. Then, a target stimulus (i.e., an arrow, an East Asian face, or a European face) was presented until response (timeout: 2000 ms), either to the left or right of the fixation point. Participants were instructed to press the “F” key if the target pointed left and the “J” key if it pointed right. They were asked to respond as quickly and accurately as possible. The word “incorrect!!” was presented for 700 ms when the incorrect key was pressed. The direction and location of the target were randomised within each experimental block. Arrow and face targets were presented in separate blocks; however, East Asian and European faces were intermixed within the same block. At the end of the experiment, participants rated the familiarity of each face on a five-point Likert scale ranging from 1 (Not at all familiar) to 5 (Extremely familiar). They were also asked to classify the ethnicity of each face as East Asian, European, or other.

### Results

Based on the criteria used in Marotta et al. (2018), RTs shorter than 200 ms (0.06%), longer than 1300 ms (0.43%), and incorrect responses (5.72%) were excluded from the RT analyses. Three European participants were not included in the analysis of the data because they did not complete the experiment. For the sake of brevity, we reported the analysis of error rates, rating scores of the familiarity, and the correct ethnicity identification rates across Experiments 1 and 2 as the supplemental data (see Supplement A).

We calculated mean RTs for each experimental condition, considering target type, congruency, and participants’ ethnicity. Mean RTs were then subjected to a three-factor ANOVA with target type (3: arrow, East Asian, and European face) and congruency (2: congruent and incongruent) as within-participant variables, and participants’ ethnicity (2: East Asian and European) as between-participant variable.

The results showed a significant main effect of target type, *F* (2, 154) = 316.26, *p* <.001, η^2^_*p*_ = 0.80, indicating that mean RTs were faster for arrow targets compared to both East Asian and European face targets (adj. *ps* < 0.01). Additionally, mean RTs were faster for European face targets than for East Asian face targets (adj. *p* <.01).

**Fig. 2:**
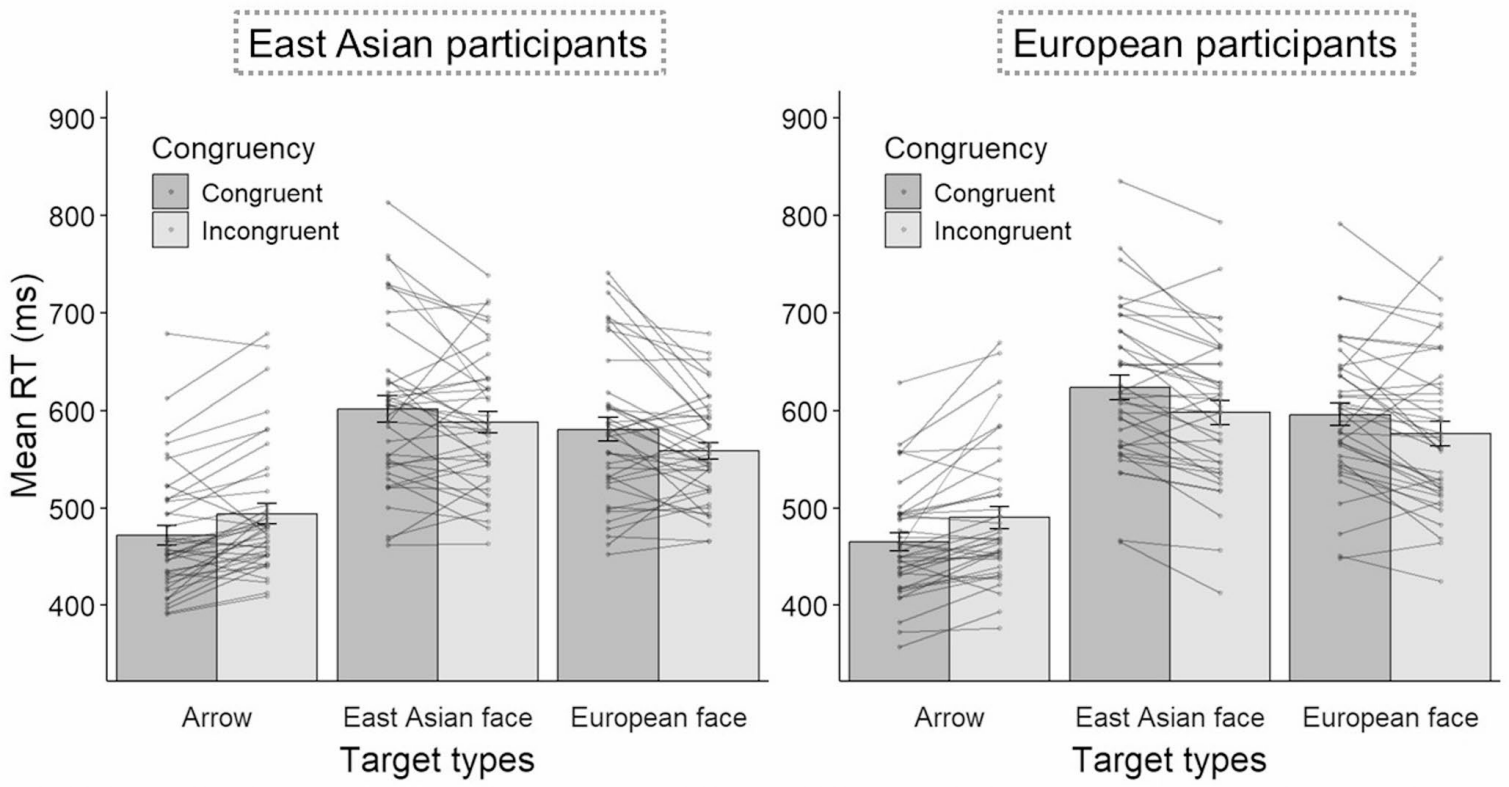
Mean RTs divided by target type, congruency, and participants’ ethnicity, in Experiment 1. Scatter plots indicate single-participant scores. Error bars represent standard errors of the mean

There was no significant interaction between target type, congruency, and participants’ ethnicity, *F* (2, 154) = 1.02, *p* =.36, η^2^_*p*_ = 0.013. Importantly, there was a significant interaction between target type and congruency, *F* (2, 154) = 38.50, *p* <.001, η^2^_*p*_ = 0.33. To clarify this interaction, we conducted a paired *t*-test for each target type. As shown in Fig. 2, RTs were shorter for congruent than incongruent trials in the arrow target, *t* (78) = − 6.03, *p* <.001, *d* = − 0.68. By contrast, RTs were shorter for the incongruent trial than for the congruent trial, both in the East Asian face targets, *t* (78) = 3.86, *p* <.001, *d* = 0.43, and in the European face targets, *t* (78) = 3.91, *p* =.007, *d* = 0.44. The other main effects or interactions were not significant, all *Fs* < 2.84, *ps* > 0.062, η^2^_*p*_ < 0.03.

### Discussion

The interaction between target type and congruency replicated previous findings (e.g., Cañadas & Lupiáñez, 2012; Marotta et al., 2018), demonstrating a classic congruency effect for arrows and the robustness of the RCE for gaze. Importantly, this interaction occurred regardless of the ethnicity of both face and participant, thus suggesting that this social variable does not appear to modulate the RCE. However, it is important to note that East Asian and European face targets were presented intermixed within the same blocks of trials. Notably, Pavan et al. (2011) and Zhang et al. (2021) reported that gaze-mediated orienting of attention for faces of two different ethnicity was modulated by this social variable according to the way these two types of face stimuli were presented (i.e., intermixed vs. blocked). We reasoned that the intermixed presentation of face stimuli adopted in Experiment 1 may have led to the emergence of possible carry-over effects, which could have, in turn, masked the potential impact of ethnicity on the RCE. Hence, we decided to run an additional experiment where everything was identical to Experiment 1, with the only exception that the two types of faces were presented in two separate blocks.

## Experiment 2

### Method

The methods used in Experiment 2 were identical to those in Experiment 1, except where noted otherwise.

#### Participants

We recruited 81 new participants, 41 of whom were East Asian (Japanese; 22 men and 19 women, *M*_age_ = 18.28 years, *SD* = 0.51), and 40 European (Italians; 22 men, 16 women, and 2 nonbinary, *M*_age_ = 26.08 years, *SD* = 2.43).

#### Procedure

In Experiment 2, the types of targets (i.e., arrow, East Asian, and European faces) were presented in separate blocks. The order of the target type was counterbalanced across the participants. The experiment comprised 192 trials: arrows (64 trials), East Asian faces (64 trials), and European faces (64 trials).

### Results

RTs shorter than 200 ms (0.02%), longer than 1300 ms (0.26%), and incorrect responses (4.82%) were excluded from the RT analyses. Three participants were not included in the analysis because they did not finish the experiment.

Mean RTs were subjected to a three-factor ANOVA with target type (arrow, East Asian, and European) and congruency (congruent and incongruent) as within-participant variables and participants’ ethnicity (East Asian and European) as between-subject variables. There was a significant main effect of target type, *F* (2, 152) = 255.15, *p* <.001, η^2^_*p*_ = 0.77, indicating that mean RTs were faster for arrow targets than for East Asian and European face targets (adj. *ps* < 0.001). Mean RTs were faster for European face targets than for East Asian face targets (adj. *p* <.01). The main effect of congruency was also significant, *F* (1, 76) = 8.36, *p* =.005, η^2^_*p*_ = 0.10.

**Fig. 3:**
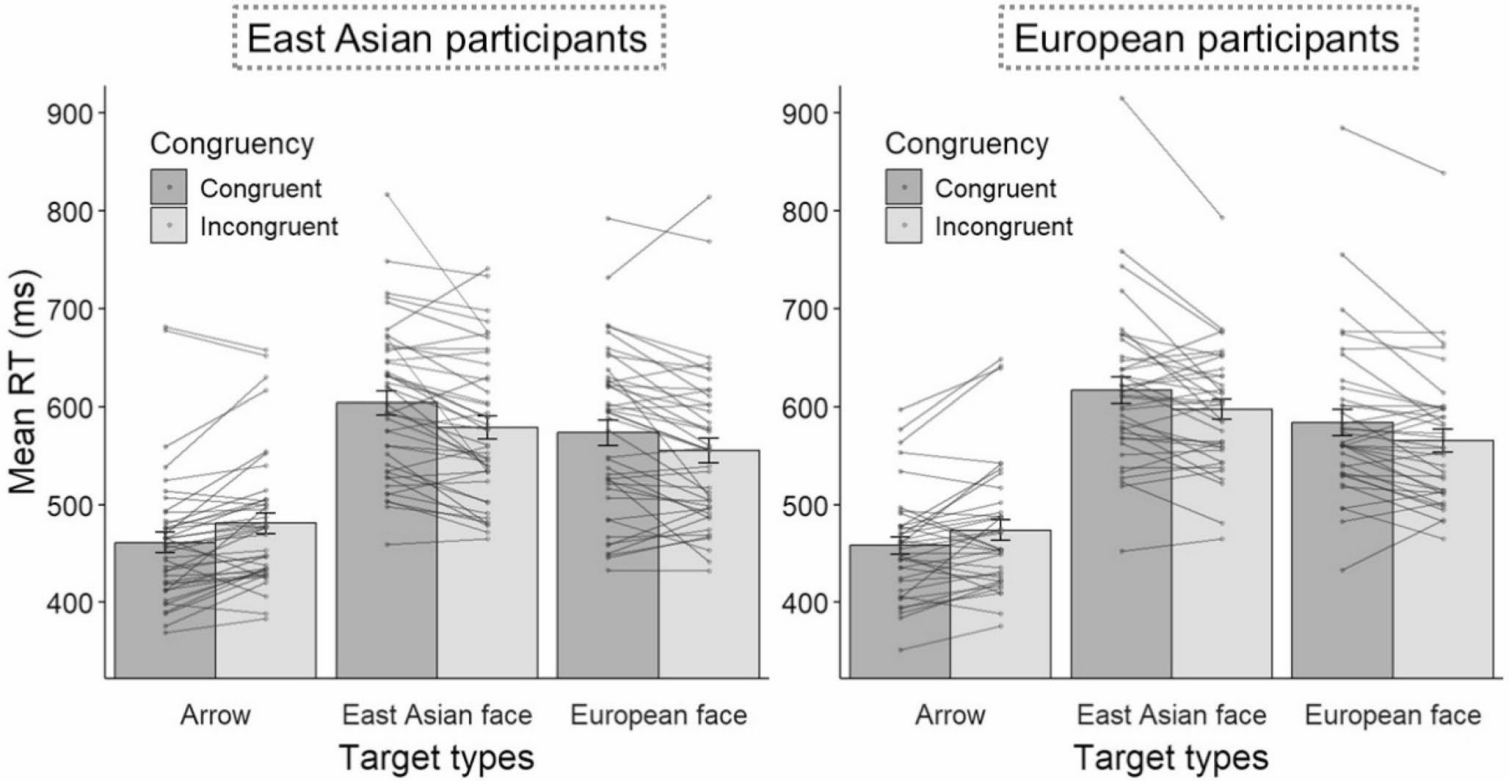
Mean RTs for the Type of Target Stimuli and Congruency in Experiment 2. Scatter plots indicate single-participant scores. Error bars represent standard errors of the mean

As shown in Fig. 3, there was a significant two-way interaction between the target type and congruency, *F* (2, 152) = 34.75, *p* <.001, η^2^_*p*_ = 0.35. We conducted paired *t*-tests for each target type to clarify this interaction. The *t*-test revealed a standard spatial congruency effect for arrow targets, *t* (77) = − 4.98, *p* <.001, *d* = − 0.56. By contrast, the RCE emerged for both East Asian, *t* (77) = 4.98, *p* <.001, *d* = 0.56, and European face targets, *t* (77) = 4.67, *p* <.001, *d* = 0.53. The other main effects or interactions were not significant, all *Fs* < 1.49, *ps* > 0.23, η^2^_*p*_ < 0.019.

## Combined analysis of the congruency effect across experiments 1 and 2

To examine the effect of participants’ ethnicity and target type on RCE across the experiments, we combined the data from the spatial Stroop tasks in Experiments 1 and 2 (*n* = 157). The congruency effect was calculated by subtracting the RTs of congruent trials from the RTs of incongruent trials. Following the approach of Wagenmakers et al. (2018), we conducted a Bayesian two-factor mixed ANOVA with target type (Arrow, East Asian, and European face) as a within-participant factor and participants’ ethnicity (East Asian and European) as a between-subjects factor. We used a Cauchy prior with a scale parameter of 0.707, which is the default setting in JASP software (JASP Team, 2016).

**Fig. 4:**
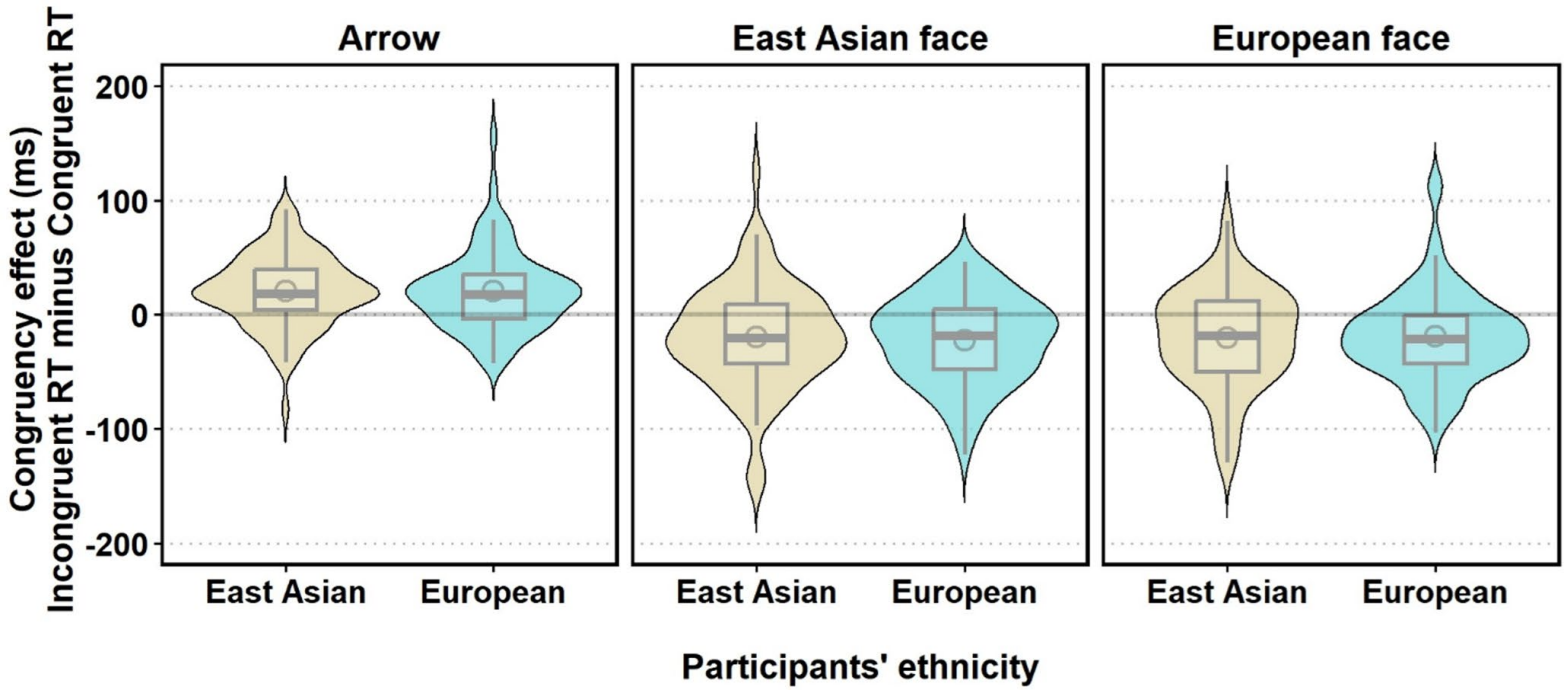
Congruency effect (ms) for Each Target Type and participants’ ethnicity across Experiments 1 and 2. The violin plot summarises the combined data of the congruency effect in Experiments 1 and 2. Box plots indicate the median and quartiles, with whiskers indicating minimum and maximum values. Gray circles in the box plot indicate the mean of the congruency effect

The result showed that the main effect of target type was significant, *F* (2, 310) = 73.33, *p* <.001, η^2^_*p*_ = 0.32, *BF*_*incl*_ = ∞, indicating that the arrows produced the standard congruency effect. As shown in Fig. 4, East Asian and European face targets produced RCE, but non-significant differences emerged in the magnitude of RCE between East Asian and European faces (adj. *p* =.76). The main effect of participants’ ethnicity, *F* (1, 155) = 0.01, *p* =.91, η^2^_*p*_ = 8.993 × 10^−5^, *BF*_*incl*_ = 0.095, and the interaction between target type and participants’ ethnicity, *F* (2, 310) = 0.16, *p* =.86, η^2^_*p*_ = 0.001, *BF*_*incl*_ = 0.024, were both not significant.

To examine the RCE both for East Asian and European face targets, we conducted a Bayesian one-sample *t*-test. The result indicated that there was strong evidence of RCE for the East Asian face (*M* = − 21, *SD* = 42, *t* (156) = − 6.22, *p* <.001, *d* = − 0.50, *BF*_10_ = 2.141 × 10^+6^) as well as for the European face (*M* = − 20, *SD* = 42, *t* (156) = − 5.94, *p* <.001, *d* = − 0.47, *BF*_10_ = 552629.343).

## Discussion

We replicated the results of Experiment 1, confirming that arrows elicited a standard congruency effect and that ethnicity did not modulate the RCE for gaze. These findings further demonstrate the robustness of this latter effect, as it emerged consistently regardless of the ethnicity of the face stimuli, the ethnicity of the participants, or the presentation context (i.e., whether faces were presented in an intermixed or blocked fashion). In addition, the combined analyses of Experiments 1 and 2 clearly indicated the robustness of the RCE for gaze, and its apparent resistance to modulation by ethnicity.

### General discussion

The present study investigated whether the RCE for gaze stimuli can be shaped by ethnic membership. In two experiments, East Asian and European participants were asked to discriminate the eye-gaze direction (left or right) of East Asian and European faces appearing leftwards or rightwards. Arrow stimuli were also included as non-social control stimuli. The results were clear-cut and in line with previous studies (e.g., Cañadas & Lupiáñez, 2012; Marotta et al., 2018): in both experiments, arrows elicited a standard congruency effect whereas eye-gaze stimuli elicited the RCE, irrespective of the participants’ ethnicity and the ethnicity of the face.

The absence of an ethnicity-based modulation of the RCE contrasts with a wide range of perceptual and attentional phenomena in the domain of social cognition, where ethnicity has been shown to exert a marked influence (e.g., Adams et al., 2010; Zhang et al., 2021; Uono & Hietanen, 2015). Further considerations are warranted based on the findings reported in the supplementary materials. First, participants in both groups accurately identified the ethnicity of the face stimuli, with over 95% accuracy. This indicates that ethnic cues were clearly recognised and processed during the task. A somewhat unexpected result, however, is that European faces were rated as more familiar than East Asian faces by both participant groups. This outcome may reflect the greater global exposure to European facial representations in media, advertising, and popular culture, which could influence perceived familiarity independently of personal experience or group membership, at least in our samples. Although this familiarity difference was present, it clearly did not modulate the pattern of the RCE.

Nevertheless, it is worth mentioning that, in both experiments, we observed that RTs were overall slower for East Asian faces than for European faces. We tentatively suggest that this finding could be attributed to differences in familiarity (see above) or specific facial characteristics, such as eye size and contrast between skin tone and the sclera, as reported by Kobayashi and Kohshima (2001). In our study, European face targets generally had larger eyes than East Asian face targets. Additionally, the contrast between the iris and the sclera/skin tone appeared to be higher in European faces, which may have enhanced the detection of gaze direction and led to faster RTs. At first sight, one might expect that such perceptual differences would also modulate the strength of the RCE, with East Asian faces (which may be perceptually more complex than European faces) producing stronger effects. However, our results showed that these RT differences did not translate into stronger RCEs. The dual-stage hypothesis (Tanaka et al., 2025) provides a useful framework to explain the relationship between perceptual complexity and the RCE. According to this model, once figure–ground segregation is achieved, response inhibition reaches a peak and remains stable for a certain period, as illustrated in delta plot analyses (see Ponce et al., 2025; Tanaka et al., 2025). Thus, while greater perceptual complexity can delay early segregation and overall response times, it does not linearly increase the magnitude of the RCE. Despite these potential RT differences, in our context the pattern of the RCE remained consistent across facial targets. This further supports the robustness of the RCE, which has been consistently replicated across multiple independent laboratories (Cañadas & Lupiáñez, 2012; Dalmaso et al., 2023; Edwards et al., 2020; Jones, 2015; Marotta et al., 2018; Tanaka et al., 2023).

In addition, one might argue that our findings contradict previous findings attributing the RCE to social factors (Aranda-Martín et al., 2022; Cañadas & Lupiáñez, 2012; Edwards et al., 2020; Hemmerich et al., 2022; Ishikawa et al., 2021, 2025; Jones, 2015; Marotta et al., 2018, 2019; Narganes‑Pineda et al., 2022; Tanaka et al., 2023). However, the evidence regarding the role of social factors, such as facial expressions and psychological disorders, remains inconclusive. Tanaka et al. (2023) found that the RCE is more pronounced for sad faces compared to happy or neutral faces. In contrast, other studies have reported stronger RCE for different facial expressions, such as happy faces (Jones, 2015; Marotta et al., 2022) or angry faces (Jones, 2015). These inconsistencies across studies suggest that factors other than social processing may be involved. One possible explanation is that variations in the RCE across facial expressions may be due to differences in eye size resulting from emotional expressions. Additionally, from a social perspective (e.g., the joint attention hypothesis), previous studies have shown that the RCE decreased for individuals with social anxiety (Ishikawa et al., 2021), while the effect was intact for those with autism spectrum disorder or attention-deficit/hyperactivity disorder (Chacón-Candia et al., 2024; Marotta et al., 2022). Other research has also suggested that the RCE is shaped by socio-cognitive development, becoming more evident as such abilities mature (Aranda-Martín et al., 2022). These studies indicate that social factors can modulate the RCE when socially attentional processes are impaired (Ishikawa et al., 2021), as in social anxiety, or when socio-cognitive development is still immature (Aranda-Martín et al., 2022). Therefore, while perceptual mechanisms such as figure–ground segregation and selective inhibition provide a baseline process underlying the RCE, it is suggested that social factors can modulate the effect in situations where impairments of these functions or their developmental trajectory are strongly linked to social factors.

The present study has some limitations. First, the East Asian and European samples differed in demographic characteristics, such as age and educational background. Nevertheless, we believe that the impact of these differences on the current findings is minimal, as the RCE was consistently observed across groups. Moreover, previous research suggests that developmental changes in the RCE are most pronounced in childhood rather than in adulthood (Aranda-Martín et al., 2022). Nevertheless, future studies could try to replicate our findings using more closely matched samples. Second, Experiments 1 and 2 were conducted online. Even if behavioural online experiments can yield reliable data (e.g., Bridges et al., 2020), this approach inevitably reduces experimental control and precludes direct monitoring of participants’ attention. To address these limitations, we instructed participants to complete the task in a quiet environment at a fixed viewing distance and implemented standard data quality checks. Importantly, the robust RCE observed across groups was consistent with prior laboratory-based studies (e.g., Cañadas & Lupiáñez, 2012; Marotta et al., 2018), suggesting that our findings are reliable despite the online format. Third, we only assessed familiarity ratings for the face stimuli. Because affective valence and arousal are key social factors that may modulate the RCE, incorporating these measures would be a valuable direction for future research.

In light of our findings, it is worth considering whether recent perceptual accounts (see Román-Caballero et al., 2021a, b; Tanaka et al., 2024, 2025) might offer a more suitable explanation of the RCE, especially in contexts where social factors (like ethnic membership) appear to play a limited role. Among these, the dual-stage hypothesis (Tanaka et al., 2025) posits that the RCE results from two sequential processes: target-background segregation as described by Román-Caballero et al. (2021a, b), and response inhibition originally discussed by Ridderinkhof, 2002a, b; Tipper, (1985). When stimuli are complex, such as faces, more effort is required to visually segregate the target, introducing a temporal delay between task-relevant and task-irrelevant codes (Hommel, 1993). This delay reduces spatial interference and allows top-down inhibitory mechanisms to operate. In incongruent trials, suppression of incorrect spatial codes facilitates responses, while in congruent trials, suppression of correct codes delays them. In our study, face stimuli likely demanded more processing effort than arrows, as indicated by longer RTs, thus triggering the inhibitory mechanisms described by the dual-stage model. Crucially, these processes were unaffected by the ethnicity of the face targets, suggesting that, at least in the present context, the RCE for gaze may have been driven more by perceptual than by social mechanisms.

## Conclusions

We investigated the possible role of ethnicity in shaping the RCE for gaze stimuli from a cross-cultural perspective. The RCE occurred irrespective of the ethnicity of the target or the participants, and the way faces were presented (i.e., intermixed vs. blocked). While previous studies provided evidence for a role of social factors in RCE (e.g., Ishikawa et al., 2025; Jones, 2015), our findings did not reveal a modulation of the effect based on ethnic group membership. This pattern may suggest that, at least under the present conditions, the RCE is relatively resilient to this social dimension and might reflect the influence of more general, possibly low-level perceptual mechanisms (see, e.g., Tanaka et al., 2025). This work contributes to the theoretical understanding of the RCE for gaze from a social perspective, suggesting that it may remain stable even across socially relevant distinctions.

## Supplementary Information

Below is the link to the electronic supplementary material.

Supplementary Material 1 (DOCX 219 KB)

## Data Availability

Behavioural data and supplemental data are available at the following link: (10.17605/OSF.IO/PR5Z2).

## Declarations

### Open practice statement

Behavioural data and supplemental data are available at the following: 10.17605/OSF.IO/PR5Z2.

### Public significance statement

When people see an arrow pointing left on the right side of a screen, they usually respond more slowly than when the arrow appears on the left, because the direction of the arrow and its position conflict. Interestingly, when the same task is performed with eye gaze instead of arrows, the opposite happens: people respond more slowly when the gaze direction and the face’s position match. This latter phenomenon is known as the *reversed congruency effect*. In our study, Japanese and Italian participants judged gaze direction from both Asian and European faces. We found that the reversed congruency effect appeared consistently, regardless of face or participant ethnicity. This suggests that, rather than being shaped by social group membership, the effect might be better explained by more general perceptual mechanisms. Our findings indicate that the way humans process others’ gaze is a robust and universal feature of visual perception.
