# Supplementary material for: Ethnicity does not matter: Comparable reversed congruency effects for gaze stimuli from same- and other-ethnicity faces
Source: Psychol Res. 2025 Oct 29;89(6):162. doi: 10.1007/s00426-025-02200-6 (PMC12568820; doi:10.1007/s00426-025-02200-6)
Supplement: Supplementary file 1 — Supplementary Material 1 (DOCX 219 KB) [file 426_2025_2200_MOESM1_ESM.docx]

Experiment 1

Results

Error rates

The mean error rates for each condition were calculated and analysed using a three-way mixed measures ANOVA with target type (arrow, East Asian, and European face) and congruency (congruent and incongruent) as within-participant variables and participants' ethnicity as between-participant variables.

A significant main effect of target type was observed, *F* (2, 154) = 5.29, *p* = .006, η^2^*_p_* = .064. The mean error rates were higher for East Asian face targets (*M* = 6.12 %, *SD* = 6.77) than for the arrow target (*M* = 4.42 %, *SD* = 4.71, *t* (78) = − 2.93, *p* = .006, *d* = − 0.33) while other comparisons showed no significant differences (all *t*s < 1.91, *p*s > .060, *d*s < 0.21). The main effect of congruency was significant, *F* (1, 77) = 11.23, *p* < .001, η^2^*_p_* = .12, indicating that the error rates were higher for the incongruent trials (*M* = 6.21 %, *SD* = 7.74) than the congruent trials (*M* = 4.40 %, *SD* = 5.93).

Furthermore, an interaction between congruency and participants’ ethnicity was significant, *F* (2, 154) = 6.29, *p* = .014, η^2^_p_ = .08. In addition, a significant interaction between target type and congruency was observed, *F* (2, 154) = 10.03, *p* < .01, η^2^_p_ = .12. To further clarify the two-way interaction, we conducted *t-*tests for each target type (arrow, East Asian, and European face). As Table 1 shows, the mean error rates of the arrow target were higher for the incongruent trials than for the congruent trials (*t* (78) = −2.93, *p* = .005, *d* = − 0.33), while no such differences were found for the East Asian (*t* (78) = 1.91, *p* = .06, *d* = − 0.21) and the European face targets (*t* (78) = 1.57, *p* = .12, *d* = 0.18). There was no significant interaction between target type, congruency, and participants’ ethnicity, *F* (2, 154) = 0.23, *p* = .79, η^2^_p_ = .003. The other main effects and interactions were not significant, all *Fs* < 2.68, *ps* > .011, η^2^*_p_* < .034

Table 1. Means and Standard Deviations of Error Rates (%) for Each Experimental Condition in Experiments 1

and 2

Familiarity rates

We calculated the mean rates of familiarity for the East Asian and European face targets. Mean rates of familiarity were subjected to mixed two-factor ANOVA with target type (East Asian and European faces) as within-participant variables and participants' ethnicity (East Asian and European) as between-participant variables.

The results showed a significant main effect of target type, *F* (1, 77) = 12.67, *p* < .001, η^2^*_p_* = .14, indicating that the mean rating of familiarity was higher for the European face target (*M* = 3.16, *SD* = 1.06) than for the East Asian face targets (*M* = 2.82, *SD* = 1.10). The other main effects or interactions were not significant, all *Fs* < 3.03, *ps* > .086, η^2^*_p_* < .04

Correct identification rates

We calculated the mean correct identification rates for face targets (East Asian and European faces) based on each participant’s ethnicity. The results indicate that East Asian participants accurately identified the ethnicity of the face targets (East Asian face: *M* = 95.63 %, *SD* = 11.16; European face: *M* = 96.25 %, *SD* = 10.67), similar to European participants (East Asian face: *M* = 96.15 %, *SD* = 10.79; European face: *M* = 89.10 %, *SD* = 21.30).

Table 2. Means and Standard Deviations of Familiarity Rates and Correct Identification Rates (%), for Each

 Experimental Condition in Experiments 1 and 2

Experiment 2

Results

Error rates

A three-way repeated-measures ANOVA examined mean error rates by target type (arrow, East Asian, European) and congruency (congruent and incongruent) as a within variable and participants’ ethnicity as a between variable. The target type exhibited a significant main effect, *F* (2, 152) = 4.64, *p* = .001, η^2^*_p_* = .058. The mean error rates were lower for arrow target (*M* = 4.61 %, *SD* = 4.38) than for the East Asian face target (*M* = 6.45 %, *SD* = 7.74, *t* (78) = −2.53, *p* = .013, *d* = − 0.28) and the European face targets (*M* = 5.93 %, *SD* = 5.24, *t* (78) = − 2.46, *p* = .016, *d* = − 0.28). The main effect of the participants’ ethnicity was significant, *F* (1, 76) = 8.66, *p* = .004, η^2^*_p_* = .10, indicating that the mean error rates were higher for the East Asian participants than for the European participants.

There was a significant interaction between congruency and participants’ ethnicity, *F* (1, 76) = 4.79, *p* = .032, η^2^*_p_* = .058. Moreover, the interaction between target type and congruency was significant, *F* (2, 152) = 6.24, *p* = .002, η^2^*_p_* = .076. To clarify the interaction between target type and congruency, we conducted a paired *t*-test for each target type. Mean error rates were lower for the congruent trial than for the incongruent trial in the arrow target, *t* (78) = − 3.86, *p* < .001, *d* = − 0.44. There were no such differences in the other pairs (all *t*s < 0.40, *p*s > .069, *d*s < 0.05). There was no significant interaction between target type, congruency, and participants’ ethnicity, *F* (2, 152) = 1.15, *p* = .32, η^2^_p_ = .015.

Familiarity rates

We calculated the mean rates of familiarity for the East Asian and European face targets. Mean rates of familiarity were subjected to a mixed two-factor ANOVA with target type (East Asian and European faces) as within-participant variables and participants' ethnicity (East Asian and European) as between-participant variables.

The results of familiarity in Experiment 2 were replicated, *F* (1, 76) = 9.32, *p* = .003, η^2^*_p_* = .11, indicating that the rating score of familiarity was higher for the European face targets (*M* = 3.00, *SD* = 0.96) than for the East Asian face targets (*M* = 2.67, *SD* = 1.34). The other main effects or interactions were not significant, all *Fs* < 1.04, *ps* > .31, η^2^*_p_* < .013.

Correct identification rates

We calculated the mean correct identification rates for face targets (East Asian and European) based on each participant’s ethnicity. East Asian participants demonstrated high accuracy in identifying the ethnicity of face targets (East Asian face: *M* = 96.15 %, *SD* = 11.05, European face: *M* = 99.36 %, *SD* = 4.11). Similarly, European participants also identified the ethnicity of face targets with high accuracy (East Asian face: *M* = 96.62 %, *SD* = 10.48; European face: *M* = 97.30 %, *SD* = 7.87).

**Combined analysis of the congruency effect (error rates) across Experiments 1 and 2**

To examine the effects of participants’ ethnicity and target type on the congruency effect across the experiments, we combined the data from the spatial Stroop tasks in Experiments 1 and 2 (*N* = 157). The congruency effect was calculated by subtracting the mean error rates of congruent trials from those of incongruent trials (Figure 1). Following the approach of Wagenmakers et al. (2018), we conducted a Bayesian two-factor mixed ANOVA with target type (arrows, East Asian faces, and European faces) as a within-participant factor and participants’ ethnicity (East Asian vs. European) as a between-subjects factor. We used a Cauchy prior with a scale parameter of 0.707, which is the default setting in JASP (JASP Team, 2016).

The results showed that the main effect of target type was significant, *F* (2, 310) = 15.92, *p* < .001, η^2^*_p_* = .05, *BF_incl_* = 84468.555, indicating that the congruency effect was higher for the arrow (*M* = 4.13 %, *SD* = 8.46) than both East Asian faces (*M* = − 0.42 %, *SD* = 8.16, *t* (156) = 5.08, *p* < .001, *d* = 0.41, *BF*_10_ = 10961.266) and the European faces (*M* = 0.59 %, *SD* = 7.31, *t* (156) = 4.01, *p* < .001, *d* = 0.32, *BF*_10_ = 167.061). There were no significant differences in the magnitude of the congruency effect between East Asian and European faces, *t* (156) = －1.38, *p* = .168, *d* =－ 0.11, *BF*_10_ = 0.226.

The main effect of participants’ ethnicity was significant, *F* (1, 155) = 10.86, *p* < .001, η^2^*_p_* = .065, *BF_incl_* = 10.288, indicating that the congruency effect was higher for the East Asian participants than for European participants. As shown in Figure 1, there was no interaction between the target type and participants’ ethnicity, *F* (2, 310) = 1.21, *p* = .30, η^2^*_p_* = .008, *BF_i_*_ncl_ = 0.024.


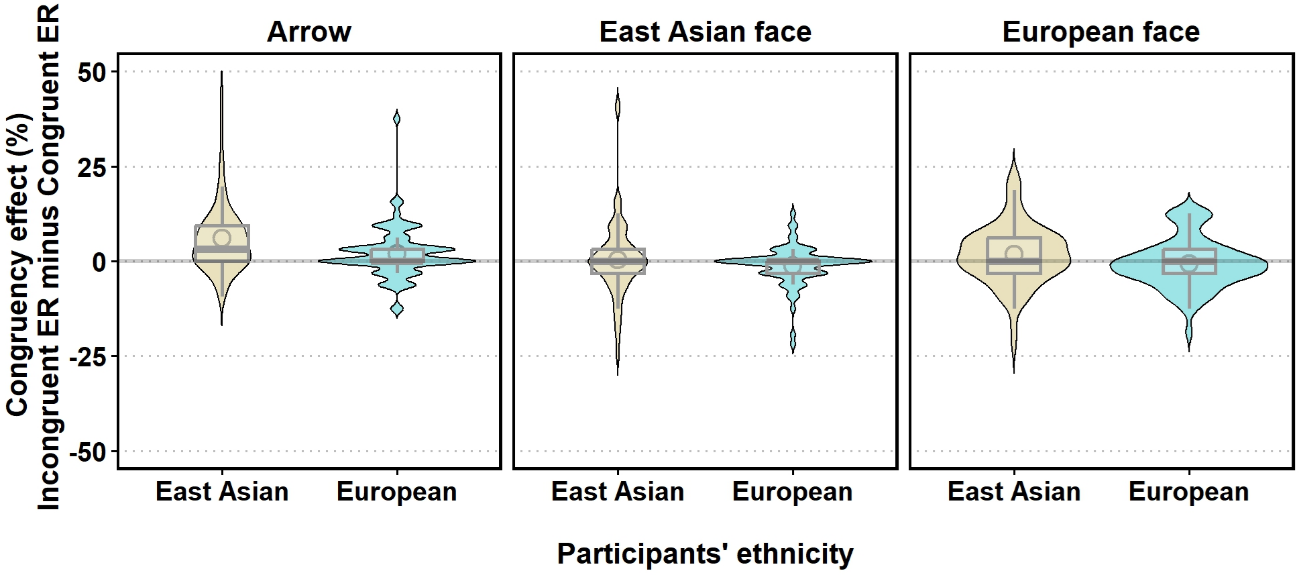
**Figure 1.** Congruency effect (%) for Each Target Type and participants’ ethnicity across Experiments 1 and 2

Note. The violin plot summarises the combined data of the congruency effect in Experiments 1 and 2. Box plots

indicate the median and quartiles, with whiskers indicating minimum and maximum values. Gray circles in the box

plots indicate the mean of the congruency effect.
